# Book Reviews

**Published:** 1916-08

**Authors:** 


### BOOK REVIEWS

**Books mentioned may be inspected at and ordered through this office. So far as possible, books received in any month will be reviewed in the issue of the second month following.**

**A Few Ophthalmic Reminders for the Busy Practitioner.** This is a booklet issued free by the Fellows Medical Mfg. Co., 26 Christopher St., N. Y.

It contains three plates in color, showing the normal fundus, and primary and secondary atrophy of the optic nerve, these plates being taken from J. M. Ball's Modern Opthalmology, published by the F. A. Davis Co. The text is based on standard authorities, and covers many points in differential diagnosis, treatment, etc. We would advise any physician who has not received this publication to apply for it.

**Skin Cancer,** Henry H. Hazen, A. B., M. D., of Washington; C. V. Mosby Co., St. Louis. 250 pages, 97 illustrations and colored frontispiece. $4.

One of the most important chapters of this work is that on the very considerable number of precancerous dermatoses, the incidental consideration of technically benign lesions being also of service in regard to prompt differentiation, and prophylaxis by early operation in doubtful cases. The general consideration of pathology is extremely valuable, all the more so because the limitations of our knowledge are frankly stated. The newer methods of treatment are considered but with due conservatism.

**Disease of the Digestive Tract and Their Treatment.** A. Everett Austin, A. M., M. D.,; C. V. Mosby Co., St. Louis. 592 pages, 85 illustrations including 10 color plates. $5.50.

This work is divided into two nearly equal parts, the first dealing with physiology, anatomy, physical and chemic examinations, dietetics and general principles of treatment; the second considering the various diseases in detail. It would perhaps be more accurate to designate the work as one on the stomach and intestine, as the oesophagus, liver and pancreas are not considered except incidentally. There is no disposition to emphasize the stomach at the expense of the intestine nor to exaggerate the importance of any one method of examination, as by the X-ray, physical and chemic methods, nor to follow a hobby in regard to dietetics or any other therapeutic means. In fact the work is well balanced and critical and represents the best work of the clinician. We quite agree with the author in his low opinion of the alizarin test, though we think that if it is especially unreliable in any one condition of acidity, it is in cases of achylia rather in those with abundant HCl. The diagnosis of hyperchlorhydria by effervescence ascribed to Fuld, was described by the editor, some years previously, as Fuld took pains to publish when his attention was called to it. Naturally, we have a somewhat higher opinion of this test than Dr. Austin, though it is of course, approximate. The estimation of acidity on an absolute basis rather than by relying entirely on degrees of deci-normal solution, is a point well taken. It seems rather doubtful, however, whether this can be definitely ascribed to E. Schuetz. It must have occurred to almost anyone engaged in gastro-enterology to determine the absolute total, either in terms of deci-normal HCl or the weight of concentrated HCl. Still, one cannot properly differentiate between hyper and hypochlorhydria, simply by estimating the absolute total of HCl present. A stomach with very scanty contents may be markedly hyperchlorhydric while one that happens to be quite full, may be distinctly hypochlorhydric, although the total amount of HCl in the latter may exceed that in the former. The colored plates of faeces, mainly from A. Schmidt, rather exaggerate the actual appearances.

---

**Elements of Active Principle Therapeutics.** J. M. French, M. D., Milford, Mass., published by The Abbott Press, Chicago, 128 pages. $0.50.

This work is arranged mainly according to diseases, i.e. by the general problems presented case by case, rather than by the potentialities of various drugs. A quarter of a century ago, the main hope of the profession for practical advance in treatment, lay in improving the materia medica, and, for the most part, by developing definite chemic substances of physiologic action, rather than relying on the crude and variable content of those substances in plants and Galenical derivatives. While many therapeutic methods have since been developed that have thrown the alkaloids in the shade, it must be acknowledged that, for practical purposes, drugs remain in a very valuable and perhaps even the most valuable part of our entire therapeutic armamentarium. That drugs should be used intelligently implies to a large degree that they should be used in definite chemic form, unmixed unless for a definite purpose, and susceptible of accurate dosage.

Thus, a work of this nature is of the highest practical value and one that should not be ignored simply because more recent means of treatment hold the center of the stage, largely because of their novelty.

---

**Progressive Medicine,** Vol. 19, No. 2, June 1, 1916. Edited by H. A. Hare and L. F. Appleman of Philadelphia, published by Lea & Febiger. Quarterly, $6 per year.

The present number (designated volume) includes Hernia by Wm. B. Coley; Surgery of the Abdomen, exclusive of hernia, by John C. A. Gerster; Gynaecology by John G. Clark; Diseases of the Blood, Diathetic and Metabolic Diseases, Thyroid, Spleen, Nutrition, and Lymphatic System, by Alfred Stengel; Ophthalmology by Edward Jackson. Gerster deals largely with war surgery. The rather cumbersome title of Stengel's division, suggests that some convenient term should be generally adopted for what is pretty well recognized as a convenient group of diseases. The work is well illustrated and, perhaps on account of personal interest, seems to us of even higher standard than that established for this quarterly review.

---

**The International Medical Annual,** 34th year, 1916 (reviewing 1915 literature), published by Wm. Wood & Co., N. Y., 735 pages, 53 page plates, 64 text illustrations, $4.

This includes a glossary of new terms, an alphabetic index of drugs with notes of new ideas, and a discussion of advances in electro-therapeutics, X-ray and radium methods, vaccine therapy, etc. A second alphabetic arrangement of titles or organs and diseases follows, comprising most of the book. The page heading suggests that only "New Treatment" is considered but many other details as of diagnosis, bacteriology, etc., are included. Special chapters follow on Naval and Military Surgery and State Medicine (or Public Health). References to last year's Annual are listed when no special advance has been chronicled since its issue. Covering almost every conceivable topic in medicine, this work is surprisingly full. Bibliographic references are given for the use of those desiring a more detailed study of any topic.

---

**Buffalo State Hospital.** 45th Annual Report for the year ending Sept. 30, 1915. J. B. Lyon Co., Albany.

This report reflects great credit upon Dr. Hurd, the Medical Superintendent and the staff. 444 patients were admitted, 330 being first admissions; 2,539 different patients were under treatment; the average population was 2,150, though the rated capacity was only 1,704. 168 died; 94 patients were discharged as cured. Sept. 30, 1914, 2,095 patients were under treatment, increased at the end of the fiscal year to 2,142, 1,894 of whom were supported by the state. The analysis of cases and reports of surgical, dental and ophthalmologic care, of pathologic studies, etc., are of interest. The actual cost was $206.87 per capita, it being estimated that farm and garden products amounted to $8,668.67 and articles made by patients to $9,674.01, 48% of the patients having been employed in useful work.

---

**Diseases of the Eye.** By George E. deSchweinitz, M. D., LL.D., Professor of Ophthalmology in the University of Pennsylvania. Eighth Edition, Thoroughly Revised and Enlarged. Octavo of 754 pages, 386 text illustrations, and seven lithographic plates. Philadelphia and London: W. B. Saunders Company, 1916. Cloth, $6.00 net; Half Morocco, $7.50 net.

We have previously reviewed earlier editions of this work, which has become a standard in the esteem of the profession, among the comprehensive and one might almost say cyclopaedic text books confined to special branches of the healing art. Except for the introduction of new matter, it was difficult in reviewing the seventh edition to realize that revision would be necessary. The eighth edition is the latest word uttered on the subject but we trust by no means the last that we shall have from this distinguished author.

---

**Disease of the Skin.** Richard L. Sutton, M. D., Kansas City; C. V. Mosby Co., St. Louis; 916 pages, 693 illustrations, 8 colored plates. $6.50.

Both in volume and elaborateness of discussion, implying wide experience and critical study, this book takes its place among the major text books. The author has made no attempt to deviate from the customary and logical arrangement of analogous works. About an eighth of the work is devoted to the general considerations of anatomy and histology, etiology, methods of diagnosis and treatment and classification. The detailed discussion of diseases, following the pathologic classification occupies the remainder of the work, up to the index, there being no appendixes. We note the following items: In spite of the tendency to include venereal diseases under dermatology and the past experience of the author as a naval surgeon, syphilis is not unduly prominent although it is given adequate space, as are tubercular lesions, yaws, leprosy, chancroid and the exanthemata.

True chromidrosis is sharply distinguished from pseudochromidrosis. Espundia, Perleche, Oroya fever and other diseases of foreign countries are well treated. Sporotrichosis cases are shown on a map of the U. S. Whenever necessary, bibliographic references are given at the end of a topic. X-ray and radium are discussed both as therapeutic measures and as causes of lesions. Sclerema and oedema neonatorum, elephantiasis, acromegaly, myxoedema, acarophobia and many other conditions of interest to the internist, are included and are treated in a broad-minded way.

---

**1915 Collected Papers of the Mayo Clinic,** Rochester, Minn. Octavo of 983 pages, 286 illustrations. Philadelphia and London: W. B. Saunders Company, 1916. Cloth, $6.00 net; Half Morocco, $7.50 net.

Without attempting to review this voluminous report in detail, we select some of the papers on unusual subjects. Luigi Durante describes 5 cases of tuberculosis of the tongue. Willigk found 2 in 1317 necropsies (sic, we are glad to note that others are using this term, autopsy really meaning something quite different); Fowler 4 in 382; Fischer 3 in 1500; Chiari 12 in 625; Adami none in 417 cases of tuberculous disease; Hamel 1 in 12,369 of pulmonary tuberculosis; von Ruck 19 in 500 clinical cases at Wynyah. The total number of cases recorded is about 258.

A. H. Sanford and G. B. New find that entamoeba buccalis is found in 14% of mouths free from gingival infection, and increasingly according to the degree of pyorrhoea but think its pathogenicity cannot be considered proved. Entamoeba buccalis, the putative germ of pyorrhoea and entamoeba histolytica of amoebic dysentery are different organisms and there is no parallelism in their presence. Before accepting ipecac alkaloids as a cure for pyorrhoea, it must be established that they actually destroy the amoebae in the mouth.

G. B. New discusses bilateral parotid tumors, classifying them according to personal experience as (unspecified) not explained or due to taking acids, or other foods causing spasm of ducts, to catarrhal inflammation (colds) etc.; syphilitic; tuberculous of which Homuth collected about 21 cases; those associated with lymphatic leukaemia or pseudoleukaemia; Mikulicz's disease; those due to local infection (not to mention mumps).

Edward C. Kendall details various experiments tending to support the idea that thyroid diseases do depend on iodin compounds, though not exactly in the ways previously conceived. The desdicated "proteins" after rejection of 80%

water and fat, are hydrolyzed by sodium hydroxid in alcohol. The acid-insoluble portion yields lauric acid, trytophan, tyrosin and other amino-acids, an indol nucleus and other N compounds and a crystalline compound ''A'' containing 60% of iodin. The acid-soluble portion is broken up artificially into physiologically inert N compounds, a reducing substance and amino-acids containing ''B'' iodin. The ''A'' iodin compound is highly toxic and seems to be directly responsible for the exophthalmic and hyperthyroid group of cases but in a way directly opposite to what has been conceived a priori and, it may be said, contradicted by analyses. That is, such thyroids contain 1/50-1/20 the amount of A iodin of normal glands instead of an excess and the iodin increases with amelioration of symptoms and regression of the gland and vice versa. The plausible explanation is that hyperthyroidism is due to diffusion of the toxic iodin compound. Conversely, in cretinism and myxoedema, while the B iodin compound seems to have no effect, the A compound in small dose (about 1/3 m.g. a day, 1/2 m. g. sometimes causing toxic symptoms) gives marked relief. A case of myxoedema was however, subjectively relieved to some degree by the B compound.

Frank C. Mann in experiments on shock and haemorrhage (after anaesthesia maintained by auto-intratracheal inhalation after producing unconsciousness) corroborates the old notion of ''bleeding to death into one's own veins.'' It was postulated that the efficient circulating blood could be withdrawn from a large artery—the femoral and that practically all left in the larger vessels could be expressed from the right auricle and vena cava, with the animal in the Trendelenberg posture. Comparative results were as follows: (averages) Normal controls: obtained from femoral 66%, from right auricle and cava 10%, total 76%, residue in tissues 24%.

Cervical cord cut: 54%, 11%, 69%, 34% residue.

Ether pushed to respiratory failure: 46%, 13%, 59%, 41% residue.

Shock by exposure of abdominal viscera: 28%, 11%, 39%, 61% residue.

Spinal cord destroyed from 6th cervical segment down: 42%, 15%, 57%, 43% residue.

Adrenalin injected after production of shock, animal bled after blood pressure had reached highest point: 38%, 8%, 46%, 54% residue.

It was pointed out that the psychic factors involved in human shock, as in railroad accidents and in traumatic practice generally, cannot be duplicated experimentally and that the maximum of shock for experimental purposes, is from abdominal exposure. Bearing this in mind, the increase in residual blood, according to degree of shock and the antagonistic action of adrenalin, are well shown.

Edward C. Rosenow and Sverre Oftedal, in a study of the aetiology and experimental production of herpes zoster, claim that it is due to a streptococcus having an elective affinity for the ganglia and posterior roots, possibly in some instances to other bacteria. In some instances, human and animal, the streptococcus is also found in the blisters.

Many other papers, of equal value, as relating to gastric and duodenal ulcer, the spleen, to surgical technic, neoplasms, etc., are not abstracted.

---

**Rules for Recovery From Tuberculosis.** Lawrason Brown, M. D., Saranac Lake, 2d edition, Lea & Febiger, 184 pages, $1.25.

This book contains rules of hygiene, especially with regard to abstinence from alcohol and tobacco, on food, fresh air, exercise, recreation, temperature, drugless control of cough, body weight, climate, care of children in households of consumptives, tuberculin, care of the mouth and a discussion of the nature of tuberculosis and of the terms cure and arrest. It is well written, contains perfectly orthodox ideas and yet we rather question the advisability of such a book for the patient, either placed in his hands by the physician as one might place a diet list, or, fortiori, if the patient should conclude that it would supplant personal medical guidance. It does not, of course, contain anything with which a well informed physician is not already familiar.

---

**Psychology of the Unconscious.** A study of the Transformations and Symbolisms of the Libido. Dr. C. G. Jung, University of Zurich, authorized translation by Beatrice M. Hinkle, M. D., N. Y. Moffatt, Yard & Co., N. Y. $4.

The author shows a wonderful familiarity with ancient religions and the work is replete with quotations, in the original or in translation, from Latin, Greek, Hebrew, Sanskrit, etc., also with poetry and descriptions of customs of primitive peoples. Psycho-analyses are also adduced from the author's abundant experience with insane and border-line nerotic cases. The translation of such a work into idiomatic English is, alone, a stupendous task. In attempting to review this book, intelligently, we are impressed with the existence of a personal mental ''blind spot.''

**Analysing Character.** Dr. Katherine M. H. Blackford and Arthur Newcomb, 3d edition, Review of Reviews Co., N. Y. 488 pages, illustrated from photographs. $3.

This book is divided into four parts: analysing character in vocational guidance; in selection of employes; in persuasion; and the principles and practice of character analysis. Tables of requirements for success in various lines of work are given. The work is very readable and interesting. The illustrations are largely of successful men in business and professional life, including politics, but include some anonymous failures. Rather by implication than assertion, character analysis is presented largely on the basis of physiognomy and cranial conformation. Excepting extreme types of cranial abnormality, we are rather sceptic as to the possibility of correct deductions from anatomic appearances; at least without very thorough training and native or acquired skill, as in any other matter. More dependence should be placed, we think, on known facts of the individual character. Perhaps it would be fairer to put this opinion in the form of a confession that the writer recognizes his own inability to judge character by inspection, without the history of the case, even to recognize in the photographs presented, the salient appearances on which it is implied that character may be judged and success or failure estimated in advance. The analogy to diagnosis of disease by inspection alone, is obvious. At intervals, two problems are brought up repeatedly, and more or less explicitly, which we think often puzzle physicians and others. To what degree shall one persevere against obstacles and discouragement and when shall one conclude that failure in a given line of work is permanent, warranting a change of vocation? How shall the physician or anyone else engaged in an occupation that involves mainly personal labor, that cannot well be conducted on a large scale, with a considerable force of assistants, conserve his energies for the real work and avoid frittering away his time and strength on details? Unfortunately, we do not find definite answers to these problems. Perhaps they are insoluble.

